# Implementation of genomic medicine for gastrointestinal tumors

**DOI:** 10.1002/ags3.12178

**Published:** 2018-06-08

**Authors:** Yoichi Furukawa

**Affiliations:** ^1^ Division of Clinical Genome Research The Institute of Medical Science The University of Tokyo Tokyo Japan

**Keywords:** genomic medicine, oncology

## Abstract

Genomic medicine is an approach to take advantage of genomic data in medical practice and health care. The advancement of sequencing technologies has enabled the determination of individual genomes as well as the genome in neoplasms. In the field of human cancer, understanding genomic alterations in tumors and variations associated with drug responses has paved the way towards the development of new drugs and personalized medicine. International collaborations of cancer genome analyses have accumulated a huge body of information about somatic mutations, and identified new driver mutations and pathways in a wide range of cancers. In particular, a growing body of evidence has shown that information about mutations in neoplasms helps to assess the efficacy and resistance of anti‐cancer drugs. Information about germline mutations associated with hereditary cancer has been shown to benefit patients by enabling early detection of their tumors and disease‐specific treatment, as well as reducing the risk for those at risk. To promote personalized medicine in a more cost‐effective and personalized way, further inter‐institutional, nationwide, and international collaboration is needed. This article summarizes the background and current situation of genomic medicine in the field of gastrointestinal tumors to help physicians and medical coworkers by assisting their better understanding of genomic medicine and strengthening their confidence of its clinical use.

## 1 INTRODUCTION

Rapid advances in DNA sequencing technology including next‐generation sequencing (NGS) have dramatically increased the power of sequencing analysis, and expanded genome science.^1^ Now we can obtain whole human genome sequencing data within a couple of days at the cost of less than $1000 per genome. Together with the progress of NGS technology, the international collaboration of cancer genome analyses has led to the comprehensive understanding of the development and progression of human neoplasms. The International Cancer Genome Consortium (ICGC), a project building a comprehensive catalogue of somatic abnormalities, started in 2008, and has recruited more than 27 000 patients for 107 projects. The Cancer Genome Atlas (TCGA), another collaborative project by the National Cancer Institute and National Human Genome Research Institute in the USA, has completed genomic analysis of 33 types of cancer from more than 11 000 patients. These projects have shown that cancer cells accumulate thousands of somatic mutations, that the number of mutations is different among cells and their tumor types, and that different and/or common driver mutations or mechanisms play a role in tumorigenesis.^1^

**Figure 1:**
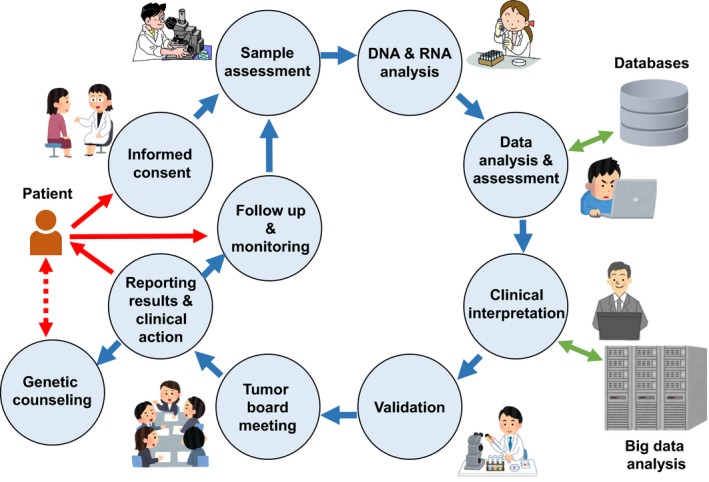
Workflow model of genomic medicine by clinical sequencing

Implementation of genomic medicine has emerged in many developed countries. In the USA, several companies and institutions launched services and projects to use somatic mutation data in personalized cancer treatment. Application of genomic medicine in clinics has been supported by the US government, as the former president, President Obama, launched the precision medicine initiative in January 2015. In 2016, the French government decided to invest 670 million euro in promoting genomic medicine and personalized medicine. Genomics England started whole genome sequencing of 100 000 patients with rare diseases and their families, as well as patients with common cancers. Although Japan was a slow starter, the Japanese government decided to support the development of systems and infrastructures for personalized medicine in the field of rare undiagnosed diseases and cancer. It is obvious that collaboration is of paramount importance for the implementation of personalized medicine. Adequate knowledge and strategies of genomic medicine need to be shared not only by the researchers and informaticians in medical and genomic studies, but also by physicians and medical coworkers in clinics, and together they need to make efforts to benefit cancer patients in a cost‐effective way (Figure 1).

The aim of this article is to overview four critical issues for the implementation of genomic medicine in gastroenterological oncology, namely: (i) personalized cancer treatment; (ii) incidental/secondary findings; (iii) assessment of cancer susceptibility; and (iv) adverse effects and resistance of anti‐cancer drugs.

## 2 APPLICATION OF NGS FOR PERSONALIZED CANCER TREATMENT

Anti‐cancer drugs have dramatically changed over the years, from drugs suppressing universal mechanisms of cell growth to those specifically targeting cancer‐driver molecules or pathways. Identification of chimeric proteins in neoplasms led to the development of a new generation of drugs such as imatinib and dasatinib for BCR‐ABL fusion proteins, and crizotinib and alectinib for ALK fusions. In addition, several receptor tyrosine kinases have become new targets of anti‐cancer drugs because their kinase activity controls the growth of some neoplastic cells. For example, gefitinib was developed to inhibit the kinase activity of epidermal growth factor receptor (EGFR) and used for the treatment of non‐small‐cell lung cancer. Later, genetic analysis disclosed that the efficacy of gefitinib was closely linked with the activating mutations in its cytoplasmic region. Now, analysis of genetic alterations in the cytoplasmic region is mandatory as a companion diagnosis for the use of EGFR inhibitors including gefitinib, erlotinib, and afatinib. It is of note that cancer cells are “addicted” to the enhanced growth signaling by the mutations in EGFR. However, therapeutic antibodies that antagonize EGFR such as cetuximab and panitumumab are used for patients with wild‐type EGFR. These antibodies do not benefit patients carrying a *RAS* mutation in their tumors because the downstream signal of EGFR is activated by *KRAS* or *NRAS* mutation. Therefore, screening of *RAS* mutations is essential for the exclusion of patients that will not benefit from the drugs. Molecular‐targeted drugs for gastrointestinal cancer, their targets, and companion diagnostics (CDx) are summarized in Table 1.

**Table 1:** Molecular targeted drugs approved for gastrointestinal malignancies in Japan

Drug	Target	Approved disease	CDx
Regorafenib	VEGFR, KIT, PDGFR, RET	CRC, GIST, HCC	
Sorafenib	Raf, VEGFR2, PDGFR	HCC, RCC, PTC	
Sunitinib	PDGFR, KIT, VEGFR, FLT3, RET	GIST, RCC, pNET	
Imatinib	PDGFR, KIT, BCR‐ABL	GIST, CML, Ph + ALL	Kit / PDGFR‐α mutation, BCR‐ABL
Everolimus	mTOR	NET, RCC, BrCa, RAML, SEGA	
Trastuzumab	HER2	Gastric Ca, BrCa	HER2(+), HER2‐amplification
Bevacizumab	VEGF	CRC, NSCLC, OvCa, Cervical Ca, Glioma, AMD	
Ramucirumab	VEGFR2	CRC, Gastric Ca, NSCLC	
Cetuximab	EGFR	CRC, HNSCC	EGFR(+), RAS (KRAS, NRAS) mutation(−)
Panitumumab	EGFR	CRC	RAS (KRAS, NRAS) mutation(−)
Nivolumab	PD‐1	Gastric Ca, Melanoma, NSCLC, RCC, HNCa, Hodgkin's lymphoma	

AMD, age‐related macular degeneration; BrCa, breast cancer; CDx, companion diagnostics; CML, chronic myeloid leukemia; CRC, colorectal cancer; EGFR, epidermal growth factor receptor; FLT3, FMS‐related tyrosine kinase 3; GIST, gastrointestinal stromal tumor; HCC, hepatocellular carcinoma; HER2, human epidermal growth factor receptor 2; HNCa, head and neck cancer; HNSCC, head and neck squamous cell carcinoma; mTOR, mammalian target of rapamycin; NET, neuroendocrine tumor; NSCLC, non‐small‐cell lung cancer; OvCa, ovarian cancer; PD‐1, programmed cell death protein 1; PDGFR, platelet‐derived growth factor receptor; Ph + ALL, Philadelphia‐positive acute lymphoblastic leukemia; pNET, pancreatic neuroendocrine tumor; PTC, papillary thyroid cancer; RAML, renal angiomyolipoma; RCC, renal cell carcinoma; SEGA, subependymal giant cell astrocytoma; VEGF, vascular endothelial growth factor; VEGFR, vascular endothelial growth factor receptor.

Development of immune checkpoint inhibitors, which are new anti‐cancer drugs that suppress deregulated immune checkpoint systems, has rapidly led to their application to various types of solid tumors including malignant melanoma, non‐small‐cell lung cancer, colorectal cancer, and other tumors. As these drugs are reactivating the immune system against cancer by hampering the immune‐escaping mechanism(s) of cancer cells, one of the factors affecting their efficacy is assumed to depend on the number of antigens expressed on the surface of tumor cells. Consistent with this view, a clinical study showed that pembrolizumab, an anti‐programmed cell death 1 (anti‐PD‐1) antibody, was effective for microsatellite instability‐high (MSI‐H) tumors, but ineffective for microsatellite instability‐stable (MSS) tumors, suggesting that a microsatellite instability (MSI) test should be a good biomarker for immune checkpoint inhibitors.^2^ Challenges are underway to discover additional candidate biomarkers.

Next‐generation sequencing has been adopted in many clinical laboratories because of its low cost and rapid speed of sequencing compared with the traditional Sanger's method. As the analysis of target molecules and/or their downstream signals leads to the discovery of predictive markers for the treatment of several anti‐cancer drugs, analysis of multiple genes is now considered to be an effective strategy to select drugs. A wide range of cancer panels such as hot‐spot mutation panels, actionable gene panels, and comprehensive gene panels have come into clinical practice. A population‐based study using a panel with 212 amplicons of 48 genes showed that clinically relevant mutations or mutations associated with human carcinogenesis were found in approximately 63% (534/854) of patients with a variety of cancers, and that actionable mutations or mutations providing information of the sensitivity or resistance to approved and preclinically available drugs were identified in about 26% of patients.^3^ Another study using a panel containing 189 amplicons of 46 genes showed that clinically relevant mutations were found in approximately 87% (296/342) of patients with mainly melanoma, non‐small‐cell lung cancer, and colorectal cancer, and that actionable mutations were identified in about 35% (122/351) of patients.^4^ Although the frequencies of mutations detected by the panels are different among studies, a good proportion of patients have benefited from panel sequencing. Notably, the advancement of analytical algorithms for whole genome and targeted sequencing data has enabled determination of copy number variations (CNV).^5^ However, the determination of slightly altered copy number including loss of heterozygosity (LOH) remains difficult especially in the cases with low tumor cell content. In addition, there are certain limitations for detecting complex structural alterations such as inversions and translocations by panel sequencing or whole exome sequencing.^6^ Naturally, the detection rate of clinically relevant point mutations and small insertions/deletions is augmented by the increase of genes in the panels, hybrid capture‐based sequencing, whole exome sequencing, and whole genome sequencing.^7^ Determination of other types of alterations such as CNV and structural variations 8 will further increase the number of patients who will benefit from the sequencing.^9^, ^10^ A recent survey showed that physicians who used the Memorial Sloan Kettering‐Integrated Mutation Profiling of Actionable Cancer Targets (MSK‐IMPACT), a next‐generation hybridization capture sequencing, changed clinical management in 21% (331/1593) of their patients according to the sequencing result.^11^ Although the physicians enrolled in the study indicated the presence of an actionable mutation in 805 of 1474 cases, expert curators judged 362 of 805 cases as actionable, suggesting that involvement of experts is crucial for the appropriate implementation of genomic medicine. In addition, clinical actionability of pathogenic variants is defined by evidence for their potential utility as therapeutic targets, but real actionability may be different depending on the country, health‐care system, insurance, accessibility to drugs, and/or various situations of individuals.

## 3 INCIDENTAL/SECONDARY FINDINGS IN NGS PANEL TESTS FOR SOMATIC MUTATIONS

As panel sequencing includes genes responsible for cancer susceptibility, it is possible to identify germline mutations in cancer tissues. In the case of analysis of tumor tissue alone, the origin of mutations may be indistinguishable from germline variants, but other clinical information may strongly enable suspicion of the presence of germline mutations. Regarding the analysis of tumor and normal pair, the origin can be easily determined. Germline mutations identified in the analysis of somatic mutation profiling may not be the initial purpose of the analysis, but some of the mutations in hereditary cancer syndromes such as Lynch syndrome are useful for the determination of treatment course for the patients. As patients with Lynch syndrome harbor a germline mutation in a gene involved in mismatch repair machinery, most of the tumors accumulate somatic mutations and are determined as MSI‐H. Furthermore, deleterious mutations in genes responsible for high‐penetrance cancer susceptibilities may be helpful for the health care of the patient's family members. The American College of Medical Genetics and Genomics (ACMG) published an updated policy statement of reporting incidental or secondary findings identified in clinical sequences and a revised list of actionable genes in 2016. The list comprises 59 medically actionable genes of 27 hereditary diseases, and includes 25 genes of 17 hereditary cancer syndromes. A study of targeted exome analysis of 202 genes, which included 18 actionable genes listed by ACMG and *PALB2*, detected likely pathogenic variants in 43 of 1000 patients enrolled in the study.^12^ Another survey tumor‐germline paired sequencing identified germline variants suspected for hereditary tumor predisposition in 19 of 439 (4.3%) cancer patients.^13^ Recently, a study reported that 182 (17.5%) of 1040 patients with advanced cancer carried clinically actionable inherited mutations detected by tumor‐normal pair sequencing, and that 97, 52, and 33 of the 182 were identified in high‐, moderate‐, and low‐penetrance genes, respectively.^14^

The latest statement of genetic and genomic testing for cancer susceptibility by the American Society of Clinical Oncology (ASCO) includes recommendations on the findings of germline mutations in multigene panel testing and whole exon/genome testing for somatic mutation profiling. The statement addresses the importance of pre‐test communication with patients about the potential for incidental and secondary germline findings, and emphasizes careful ascertainment of patient preferences regarding the receipt of germline information. It additionally gives a caution about the quality assurance and clinical validity of the germline variants.^15^ As the germline mutations associated with hereditary diseases affect not only the treatment of patients but also health care of the family members, report of germline information should be carried out by a specialist such as a genetic counselor or expert physician. However, the statement does not assert the obligation of searching actively for incidental or secondary findings in the tests for somatic mutations because the cost and efforts of germline analysis may hamper the primary purpose of identifying actionable driver mutation(s) for the patient's cancer care.

## 4 ASSESSMENT OF CANCER SUSCEPTIBILITY FOR EVIDENCE‐BASED HEALTH CARE

Prevention and early detection are two of the most effective strategies to avoid death from malignant neoplasms. Family history is important information for the assessment but the determination of deleterious mutation in the responsible genes will give us more beneficial information for personalized health care. For example, identification of germline mutations in the genes susceptible for hereditary cancer such as Lynch syndrome is useful for the treatment of patients as described in the previous section. In addition, germline information for the susceptibility to hereditary cancer with high penetrance should be beneficial for the health care of the patient's family members. Therefore, for patients with suspected predisposition to familial or hereditary cancer, the physician in charge should consult specialists of hereditary cancer or genetic counselors. Enrolment into the surveillance programs of individuals at risk was proven to decrease their risk of death from disease‐associated cancer.^16^, ^17^ Assessment of cancer susceptibility is necessary for consideration of the treatment, prevention and surveillance. For patients with Lynch syndrome, a hereditary disease that predisposes individuals to various types of human tumors including colorectal cancer, endometrial cancer, urothelial cancer, tumors of the small intestine, and gastric cancer, National Comprehensive Cancer Network (NCCN) recommends surveillance not only of the colon but also of other organs that may give rise to Lynch syndrome‐related tumors in the genetic/familial high‐risk assessment for colorectal cancer version 2, 2017. In addition, consideration of near‐total colectomy, hysterectomy, and/or bilateral salpingo‐oophorectomy is needed as an option at the diagnosis of newly developed colorectal cancer. Interventions that can reduce the development of cancer have been proposed, and several guidelines are provided and updated for hereditary cancers. Risk reduction interventions should be carried out on the basis of the patients' informed choice, after communication with genetic counselors and prudent consideration.

The advantages of NGS have accelerated the application of multiplex or multigene panels for the assessment of cancer susceptibility. It was reported that NGS successfully identified 15 877 of the 15 878 variants that were identified by the traditional Sanger's method.^18^ NGS allows for the simultaneous testing of many genes, including high‐penetrance genes with established clinical utility, and moderate‐penetrance genes the clinical utility of which is less clearly established. It is noteworthy that the multiplex and multi‐gene panel test may not find a deleterious mutation in the gene anticipated by the subject's family history but may find a mutation in another unanticipated high‐penetrance gene.^19^ More importantly, although tests for high‐penetrance genes with a relative risk greater than four compared to the general population may provide practical management strategies for the patient's health care, the result of moderate‐penetrance genes with a relative risk of between two and four should be carefully considered before reporting the results because management of individuals with the mutations may not yet be established. Therefore, ASCO affirms that evaluation of genes of established clinical utility is sufficient in the search for possible explanations for a patient's personal or family history of cancer. Moreover, it should be noted that in a substantial number of cases, genetic tests using multiplex or multi‐gene panel identify variants of unknown significance (VUS) that may or may not affect the function of the gene product. The number of VUS depends on the genes analyzed and increases according to the number of genes tested. In particular, the number is dramatically augmented when moderate‐penetrance genes are included in the test because rare variants in the moderate‐penetrance genes are difficult to evaluate correctly. As the report of VUS will not change clinical management but will bring confusion and psychological stress to subjects, information on VUS is recommended to be reserved until their clinical utility has been determined. Clinical studies on the management of patients with mutation(s) in moderate‐penetrance genes, and the evaluation of VUS through international collaborations are of great necessity for the development of evidence‐based practice in the care of individuals with cancer susceptibility.

## 5 ADVERSE EFFECTS AND RESISTANCE OF ANTI‐CANCER DRUGS

Adverse effects are one of the major problems that hamper the use of anti‐cancer drugs in clinical practice. Studies have been focused on genetic polymorphisms significantly altering the pharmacokinetics of anti‐cancer drugs. The polymorphisms include drug‐metabolizing enzymes and transporters because they affect the uptake, metabolism, and elimination of the drugs. Among them, polymorphisms in *UGT1A1*,* DPYD*, and *TPMT* genes have been shown to affect metabolism and detoxification of irinotecan, 5‐fluorouracil (5‐FU), and mercaptopurine, respectively.^20^ Regarding the metabolism of irinotecan, a camptothecin derivative, UDP‐glucuronosyltransferase 1A1 (UGT1A1) plays a vital role in its detoxification. As the polymorphisms (UGT1A1*28 and UGT1A1*6) are closely related to severe adverse effects of irinotecan, the dosage of irinotecan is to be decided according to the UGT1A1 polymorphisms. 5‐FU and capecitabine are frequently prescribed for the treatment of cancers in the gastrointestinal tract, breast, and head and neck. These drugs are mainly catabolized by dihydropyrimidine dehydrogenase (DPD), and a number of variants such as IVS14 + 1G > A (DPYD*2A), c.1679T > G (DPYD*13), and 2846A > T in the *DPYD* gene encoding DPD are associated with their clearance.^21^ The Clinical Pharmacogenetics Implementation Consortium (CPIC) recommends consideration of other drugs for poor metabolizers or patients carrying two copies of non‐functional DPYD variants, and reduction of fluorouracil, capecitabine, and tegafur in the starting dose for intermediate metabolizers or patients carrying a combination of a functional and a non‐functional variant.^22^ The FDA (Food and Drug Administration)‐approved drug labels for fluorouracil and capecitabine include information about the potential for severe toxicity in patients with DPD deficiency, but the labels at present do not mention testing for variants of *DPYD* or activity of DPD. Although additional studies on the association between genotype and toxicity are needed, DPYD genotype‐guided selection and/or dose adjustments will be a standard of care in the near future. Thiopurine methyltransferase (TPMT) is an enzyme involved in the metabolism of 6‐mercaptopurine (6‐MP) that is used for the treatment of acute lymphoblastic leukemia. A number of variants are associated with the enzyme activity. Among them, three variants, TPMT*2, TMPT*3A, and TMPT*3C, have been shown to significantly decrease enzyme activity in the Caucasian population.^23^, ^24^, ^25^ However, information on polymorphisms associated with the adverse effects of other anti‐cancer drugs is limited.

Drug resistance is another critical problem to be resolved in clinical practice. Studies have shown that multiple mechanisms underlie drug resistance. These mechanisms include drug detoxification, alteration(s) of drug targets, activation of other signaling pathways bypassing the target molecule(s), reduced susceptibility to apoptosis and cell death, reflux of the drugs, alteration of intrinsic characteristics (such as epithelial–mesenchymal transition [EMT] and histological conversion), and/or selection of resistant cells in the heterogeneous tumor population.^26^ One of the well‐characterized mechanisms is acquisition of resistant mutations; for example, acquisition of the T790M mutation in EGFR leads to resistance to gefitinib.^27^ Regarding imatinib which suppresses kinase activities of ABL, cKIT, and platelet‐derived growth factor receptor alpha (PDGFRA), resistant mutations in gastrointestinal stromal tumors include L576P, V654A, T670I, or Y823D in *cKIT*, and D842V or D846V in *PDGFRA*.^28^ These data are useful for designing personalized chemotherapy and the development of new drugs effective for tumors with resistant mutations. In addition to secondary mutations in the drug targets, mutations in the downstream signaling molecules of the target also play a vital role in tolerance. It has been reported that colorectal cancers become resistant to anti‐EGFR treatment with cetuximab by the acquisition of activating mutations in *KRAS*.^29^

These pieces of information are useful to understand the mechanisms of resistance in refractory tumors, and may be applicable for selection of other drugs or modalities that are effective in secondary mutation. However, in many cases, resistance originates from multiple non‐mutational, non‐genetic mechanisms.^30^ Therefore, incorporation of transcriptome, epigenome, proteome, and metabolome into genomic information is needed for precise assessment of resistant mechanism(s). Integration of these data together with information on drug efficacy, toxicity, and resistance will be a vital challenge for the promising development of specific and effective precision medicine.^31^

## 6 TUMOR HETEROGENEITY AND EVOLUTION

Current assessment of the tumor genome is mainly carried out using tissues obtained from biopsies or surgical specimens. Importantly, recent studies have clarified that tumors have subclones or intertumor/intratumor heterogeneity because tumor cells have been accumulating new mutations during their development and progression.^32^ In the analysis of tumor tissues, we should keep in mind that we are determining the tumor genome in the region(s) or specimen at the time of biopsy or surgical operation. Driver mutations that occurred early in tumorigenesis can be shared in the subclones, but late events acquired in one subclone are not shared in the other subclones. Occasionally, different mutations in the same driver gene(s) or pathway(s) are identified in different subclones,^32^ suggesting that some selection or oncogenic pressure may be involved in tumor evolution. In the analysis of tumor genome, heterogeneity results in sampling bias, and may hamper correct decision‐making for therapeutic strategy. Analysis of multiple regions in the tumor(s) and longitudinal analysis may facilitate detection of tumor evolution. However, sampling of metastasized or recurrent tumors is practically difficult. Although there is currently no technology to analyze tumor heterogeneity comprehensively, repeated analysis of circulating tumor cells and/or cell‐free DNA may be helpful to clarify at least some of the spatiotemporal changes in the tumor(s).

Notably, subclones may acquire additional changes over time during progression or in response to treatment. For example, treatment of patients with non‐small‐cell lung cancer carrying an activating mutation in EGFR using EGFR‐tyrosine kinase inhibitors (EGFR‐TKI) led to the expansion of a subclone carrying EGFR T790M, a mutation resistant to EGFR‐TKI.^33^ Treatment of colorectal cancer with panitumumab, an anti‐EGFR antibody, induced resistance to the drug in the tumors through the acquisition of *KRAS* mutations.^29^, ^34^ Therefore, it is important to consider that cancer is a disease with multiple genetic alterations in different subclones, and that they are changeable by the acquisition of new mutation(s) in response to treatment. Understanding of tumor evolution and changes in response to treatments should contribute to precision medicine through the identification of rational strategies that suppress the growing subclone(s) as well as the emergence of resistant subclone(s).

## 7 CHALLENGES FOR BETTER PRECISION ONCOLOGY

Although cost‐effectiveness of genetic testing is an important matter of concern from the financial point of view, it will not be discussed here because it is beyond the scope of this review. Current challenges for medical oncology include the interpretation of variations in the individual genome and in the cancer genome. Larger collaborations and data repositories are needed for the assessment of less common variants, which otherwise might elude statistical analysis. Accumulating genomic data are rapidly expanding and now overflowing beyond our recognition. In the near future, we will obtain epigenomic, proteomic, metabolomic, and microbiota information that will require clinical interpretation. Therefore, it is a matter of pressing necessity to develop integrative databases and analytical systems for these big data. Several institutions and academic foundations started to share genomic and clinical information. For example, the American Association for Cancer Research (AACR) launched a collaborative project named GENIE (Genomics Evidence Neoplasia Information Exchange) to promote personalized health care of patients with a wide range of neoplasms. The latter challenge requires rapid‐learning computer‐based systems in clinical oncology. New technologies and methodologies such as circulating tumor cells and free circulating tumor DNA will widen the approaches for precision medicine. More importantly, the development of applicable drugs for a wide range of driver alterations is essential for the promotion of genomic medicine. The number of current molecular targeted drugs for gastrointestinal tumors is limited compared with those for hematological malignancies and lung cancer.

In conclusion, because information and technology are rapidly expanding in the field of genomic analyses, gastroenterologists need to collaborate with oncologists, geneticists, pharmacologists, computational biologists, and bioinformaticians for the implementation of genomic medicine in gastrointestinal malignancies.

## DISCLOSURE

Conflicts of Interest: Author declares no conflicts of interest for this article.
